# Transcriptomic responses to shifts in light and nitrogen in two congeneric diatom species

**DOI:** 10.3389/fmicb.2024.1437274

**Published:** 2024-08-14

**Authors:** Xiao Ma, Zhen Qin, Kevin B. Johnson, L. Holly Sweat, Sheng Dai, Gang Li, Chaolun Li

**Affiliations:** ^1^Key Laboratory of Tropical Marine Bio-Resources and Ecology, South China Sea Institute of Oceanology, Chinese Academy of Sciences, Guangzhou, China; ^2^Guangdong Province Key Laboratory of Applied Marine Biology, Guangzhou, China; ^3^Department of Biological Sciences, College of Science and Mathematics, Tarleton State University, Stephenville, TX, United States; ^4^Smithsonian Marine Station, Fort Pierce, FL, United States; ^5^Daya Bay Marine Biology Research Station, South China Sea Institute of Oceanology, Chinese Academy of Sciences, Shenzhen, China; ^6^University of Chinese Academy of Sciences, Beijing, China

**Keywords:** transcriptomics, light, nitrogen, cell size, *Thalassiosira*

## Abstract

Light and nitrogen availability are basic requirements for photosynthesis. Changing in light intensity and nitrogen concentration may require adaptive physiological and life process changes in phytoplankton cells. Our previous study demonstrated that two *Thalassiosira* species exhibited, respectively, distinctive physiological responses to light and nitrogen stresses. Transcriptomic analyses were employed to investigate the mechanisms behind the different physiological responses observed in two diatom species of the genus *Thalassiosira*. The results indicate that the congeneric species are different in their cellular responses to the same shifting light and nitrogen conditions. When conditions changed to high light with low nitrate (HLLN), the large-celled *T. punctigera* was photodamaged. Thus, the photosynthesis pathway and carbon fixation related genes were significantly down-regulated. In contrast, the small-celled *T. pseudonana* sacrificed cellular processes, especially amino acid metabolisms, to overcome the photodamage. When changing to high light with high nitrate (HLHN) conditions, the additional nitrogen appeared to compensate for the photodamage in the large-celled *T. punctigera*, with the tricarboxylic acid cycle (TCA cycle) and carbon fixation significantly boosted. Consequently, the growth rate of *T. punctigera* increased, which suggest that the larger-celled species is adapted for forming post-storm algal blooms. The impact of high light stress on the small-celled *T. pseudonana* was not mitigated by elevated nitrate levels, and photodamage persisted.

## Introduction

Diatoms, considered one of the most diverse and ecologically important phytoplankton groups, contribute around 20% of global primary productivity (Malviya et al., 2016). The genus *Thalassiosira* comprises the of the centric diatoms with more than 100 species, including many with cosmopolitan distributions (Smith and Johnson, 1996; Malviya et al., 2016; Park et al., 2016). The species in the genus were frequently reported as bloom-forming diatoms (Brodie et al., 2010; Okhapkin et al., 2016; Sidabutar et al., 2021; Kang et al., 2022; Ma et al., 2022). The cell sizes of species in *Thalassiosira* range from less than 2 μm to over 200 μm, and species’ cell volumes vary over more than nine orders of magnitude (von Dassow et al., 2008; Litchman et al., 2009). Cell size and volume strongly impact phytoplankton ecological functions, such as light absorption, nutrient uptake, metabolic requirements and interactions with grazers (von Dassow et al., 2008; Ma et al., 2021). Field observations have shown that large-sized phytoplankton tend to form algal blooms after tropical cyclones. For example, the algal bloom after Lekima Typhoon were mainly composed of *Pseudo-nitzschia* spp., *Noctiluca scintillans* and *Coscinodiscus* spp., which were much larger than the pre-typhoon dominant diatom species *Chaetoceros* spp. (Jiang et al., 2022). Light and nitrogen availability are essential drivers impacting phytoplankton physiology, life processes, and community composition (Berges and Falkowski, 1998; Solovchenko et al., 2008; Cointet et al., 2019).

**Figure 1:**
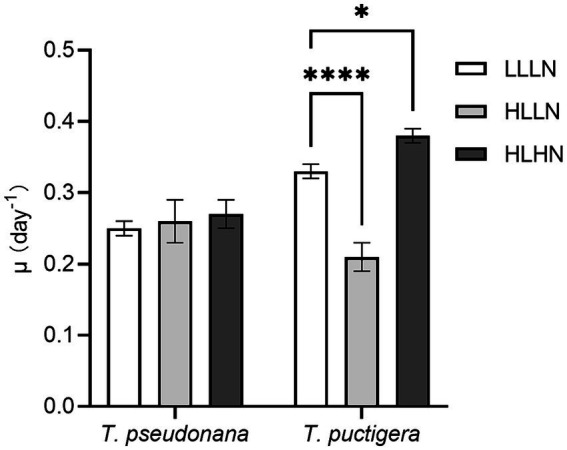
Cell specific growth rates (μ) of the small-celled *Thalassiosira pseudonana* and the large-celled *T. punctigera* under low light low nitrogen (LLLN), high light low nitrogen (HLLN) and high light high nitrogen (HLHN) culture conditions. Statistical analysis was conducted with two-way ANOVA test with *post-hoc* Dunnett’s multiple comparisons. * indicates *p* < 0.05, **** indicate *p* < 0.0001. This figure was modified from our previous study: Qin et al. (2021).

In our previous study, the small-celled diatom species *Thalassiosira pseudonana* (~40 μm^3^) and the large-celled *T. punctigera* (~300,000 μm^3^) were selected, and their physiological characteristics were compared under different light and nitrogen conditions (Qin et al., 2021). Experimental treatments monitored diatom growth under three environmental scenarios: (1) subsurface diatom populations in the field with low light and low nitrogen conditions (LLLN, 35 μmol photons m^−2^ s^−1^, ~10 μM N in media); (2) diatom populations brought near the surface intermittently and exposed to high light intensity but low nitrogen conditions (HLLN, 250 μmol photons m^−2^ s^−1^, ~10 μM N in media); and (3) diatom populations in a strongly mixed surface water column, such as immediately following tropical cyclone conditions with exposure to elevated light and nitrogen conditions (HLHN, 250 μmol photons m^−2^ s^−1^, ~800 μM N in media) (Qin et al., 2021). The growth rates of the small-celled *T. pseudonana* and large-celled *T. punctigera* under LLLN conditions were 0.25 ± 0.01 d^−1^ and 0.33 ± 0.01 d^−1^, respectively (Figure 1). When they were transferred to HLLN conditions, the growth rate of the small-celled *T. pseudonana* was not affected, but that of the large-celled *T. punctigera* significantly decreased (*p* < 0.001) (Figure 1). On the other hand, when they were transferred to HLHN conditions, the growth rate of the large-celled *T. punctigera* significantly increased (*p* < 0.05), while that of the small-celled *T. pseudonana* remained unchanged (Figure 1). The two congeneric diatom species exhibited disparate physiological responses to light and nitrogen conditions. The small-celled *T. pseudonana* adjusted to the HLLN conditions rapidly, suggesting that it could maintain stable growth if brought to the surface. On the other hand, the large-celled *T. punctigera* appear to prefer HLHN conditions and may be more likely to form algal blooms after tropical cyclones. To answer the question why the two diatom species that belong to the same genus displayed such different physiological responses to the environmental changes, transcriptomic analyses were conducted on diatoms living under the previously described light and nitrate conditions to better understand the respective unique physiochemical responses of these congeneric species.

## Materials and methods

### Culture protocol

Both *T. pseudonana* (CCMP 1335) and *T. punctigera* (CCAP 1085/19), were semi-continuously cultured in 0.2 μm-filtered and sterilized natural seawater (fsw) at 18°C in 175 mL Thermo Scientific™ Nunc™ EasYFlask™ Cell Culture Flasks with integral 0.2 μm PTFE membrane cap. The seawater was collected from the open ocean of the South China Sea where total nitrogen content was less than 1.0 μM. To get enough algal cells for RNA extraction, the cultures were batch cultured to the highest biomass density possible before the culture enters the stationary phase, and then semi-continuously cultured and maintained at these high densities. A semi-continuous mode of cultivation implies that half of the culture was discarded at periodic time intervals (i.e., every cell replication generation in this study) while half new medium was added. Thus, the algal density was maintained at a stationary high level continuously. Indeed, the cell densities of *T. pseudonana* and *T. punctigera* were maintained at (6.00 ± 3.15) × 10^5^ and 1,385 ± 659 cells mL^−1^ for at least 6 cell replication generations, respectively. The procedures were conducted in a horizontal laminar flow clean bench. In contrast to a constant supply of nutrients under natural conditions, the nutrients in the media is quickly depleted in laboratory cultures, especially under these high algal density conditions (Li and Campbell, 2017). In this study, the Guillard’s algal culture medium was used with nitrate concentrations set to 88.2 μM (f/20) for LN and 882 μM (f/2) for HN treatments. Our previous study has demonstrated that the final nitrate concentration in the media at the end of one cultivation cycle was approximately equal to the added nitrate concentration subtract algal cellular N content (Li and Campbell, 2017). The cellular N content of *T. pseudonana* and *T. punctigera* were 1.82 ± 0.29 and 808.23 ± 180.96 pg N cell^−1^ under similar culture conditions, respectively (Li and Campbell, 2017). Subtracting the cellular N content, the nitrate concentrations in the culture media were approximately 10 μM in LN treatments and 800 μM in HN treatments. Thus, the LN treatments were nitrogen limited, while HN treatments were nitrogen excessive in the culture media when the algal samples were collected (Qin et al., 2021). During cultivation, all flasks were randomly distributed in a plant growth chamber (Zhichu, Shanghai, China) and manually shaken 2 to 3 times per day. The light in the chamber was provided by a panel of fluorescent tubes, which were automatically turned on at 7:00 a.m. and turned off at 7:00 p.m. to maintain a 12:12 h light:dark cycle. The light intensity was measured with a micro-spherical quantum sensor (ULM-500, Walz, Effeltrich, Germany) in a flask filled with seawater. Three replicates (*n* = 3) were cultured for each treatment.

### Light and nitrogen shift experiment and sample collection

For the light shift experiment, the *T. pseudonana* and *T. punctigera* were pre-cultured at LLLN condition (LL: 35 μmol photons m^−2^ s^−1^, close to the bottom of the euphotic zone) for 6 cell replication generations. Our previous study demonstrated that the diatom cultures achieved a steady state in terms of maximum photochemical quantum yield (F_V_/F_M_) of photosystem II (PSII) after 6 generations (Qin et al., 2021). Cultures acclimated to LLLN were then shifted to HLLN or HLHN condition (HL: 250 μmol photons m^−2^ s^−1^, close to the 10% of the upper euphotic zone). Cell concentrations of the LLLN inoculations were (6.00 ± 3.15) × 10^5^ and 1,385 ± 659 cells mL^−1^ for *T. pseudonana* and *T. punctigera*, respectively. They were transferred to HLLN or HLHN and cultivated semi-continuously for at least another 6 generations, and three flasks were continuously maintained under LLLN as control. At the end of cultivation, 30 mL of algae cultures were removed from each treatment at 9:00 AM and centrifuged at 5,000 *g* for 10 min at 18°C. The precipitated cells were then flash-frozen in liquid nitrogen and stored at −80°C for later transcriptomic analysis.

### Transcriptomic sequencing and data analysis

Total RNA was extracted and purified using Direct-zol RNA Miniprep Kits (ZYMO Research R2050) following the manufacturer’s instructions. The mRNA was isolated by Genewiz Company (Guangzhou, China) using NEBNext Poly(A) mRNA Magnetic Isolation Module (New England Biolabs, Ipswich, MA, United States), then underwent library preparation with a NEBNext® Ultra™ RNA Library Prep Kit for Illumina® following the manufacturer’s recommendations. The library was sequenced on an Illumina Hiseq platform.

Transcriptomic analyses were conducted using the SqueezeMeta v1.1.0 pipeline (Tamames and Puente-Sánchez, 2019), which has been used in similar transcriptomic analyses (Xia et al., 2020; Maslać et al., 2022). To conduct unbiased comparison between *T. pseudonana* and *T. punctigera*, the bioinformatic analysis pipeline for the both diatom species was the same with no reference genome. Transcriptomic sequences were filtered using Trimmomatic. Short contigs (<250 bps) were removed using Prinseq, then the contigs were merged using Minimus2. The open reading frames (ORFs) were predicted and similarity searches were conducted using Diamond against GenBank, eggNOG, KEGG and CAZyDB databases with default settings. The HMM homology searches were done by HMMER3 for the Pfam database. The abundance of each ORF was calculated as transcripts per million (TPM) = rg × rl × 106 / cl × T, where rg indicates the reads mapped to gene g, rl is read length, cl is the coding sequence length, and T is the sum of rg × rl / cl for all genes. The differential expressed genes (DEGs) among treatments were identified using the DESeq2 v1.32.0 package with |log_2_(fold change)| >1 and *p*-value <0.05. The KEGG pathway enrichment analyses were conducted using r package pathfindR with a *Q*-value <0.05 (*Q*-value is the minimum false discovery rate at which the pathway is deemed significantly enriched) (Ulgen et al., 2019). Separate enrichment analyses of pathways were conducted for up- and down-regulated genes (Hong et al., 2014). The sequences obtained in this study have been deposited in the NCBI Sequence Read Archive (SRA) under BioProject ID: PRJNA1061770.

## Results

### Transcriptomic sequencing statistics

The numbers of paired-end raw reads sequenced within each of the samples ranged from 22.67 to 35.53 million, resulting in 38,950–52,459 ORFs (Supplementary Table S1). In total, 6,093 and 5,329 genes were identified from the transcriptomes, and they were assigned to 133 and 138 KEGG pathways (level three) for *T. pseudonana* and *T. punctigera*, respectively.

### DEGs and enriched KEGG pathways

**Figure 2:**
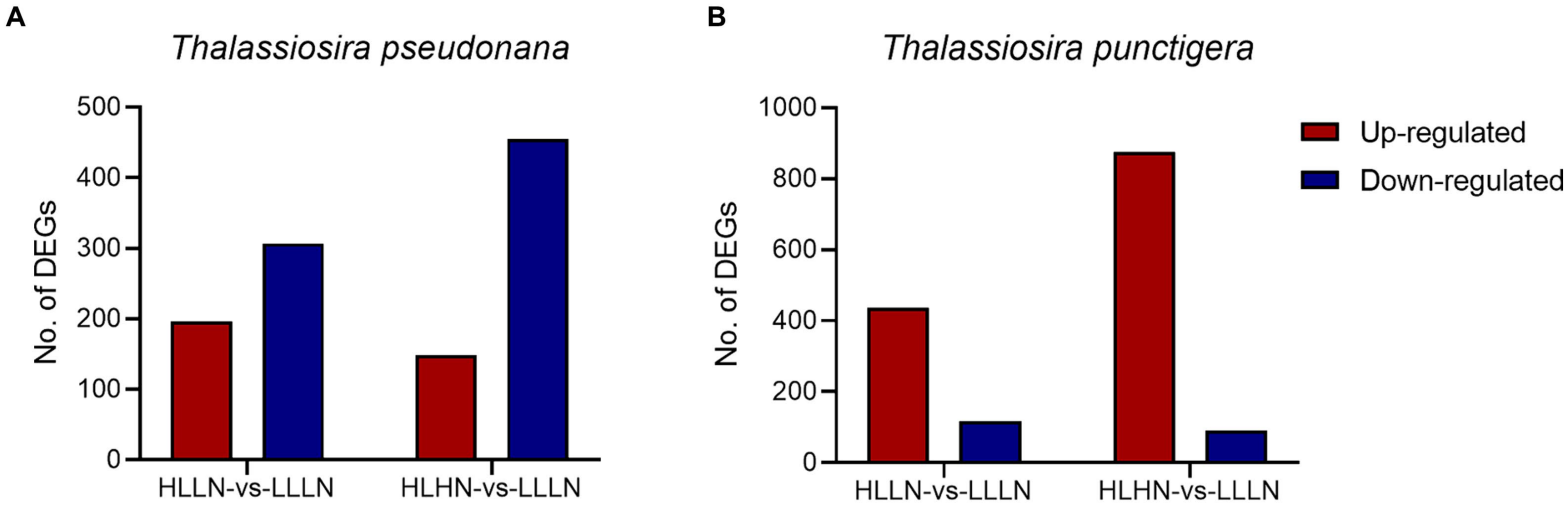
Number of differentially expressed genes (DEGs) (|log_2_(fold change)| >1, *p* < 0.05) under high light low nitrogen (HLLN) vs. low light low nitrogen (LLLN) and high light high nitrogen (HLHN) vs. low light low nitrogen (LLLN) culture conditions for **(A)** *Thalassiosira pseudonana* and **(B)** *T. punctigera*. “a vs. b” means a compared to b.

**Figure 3:**
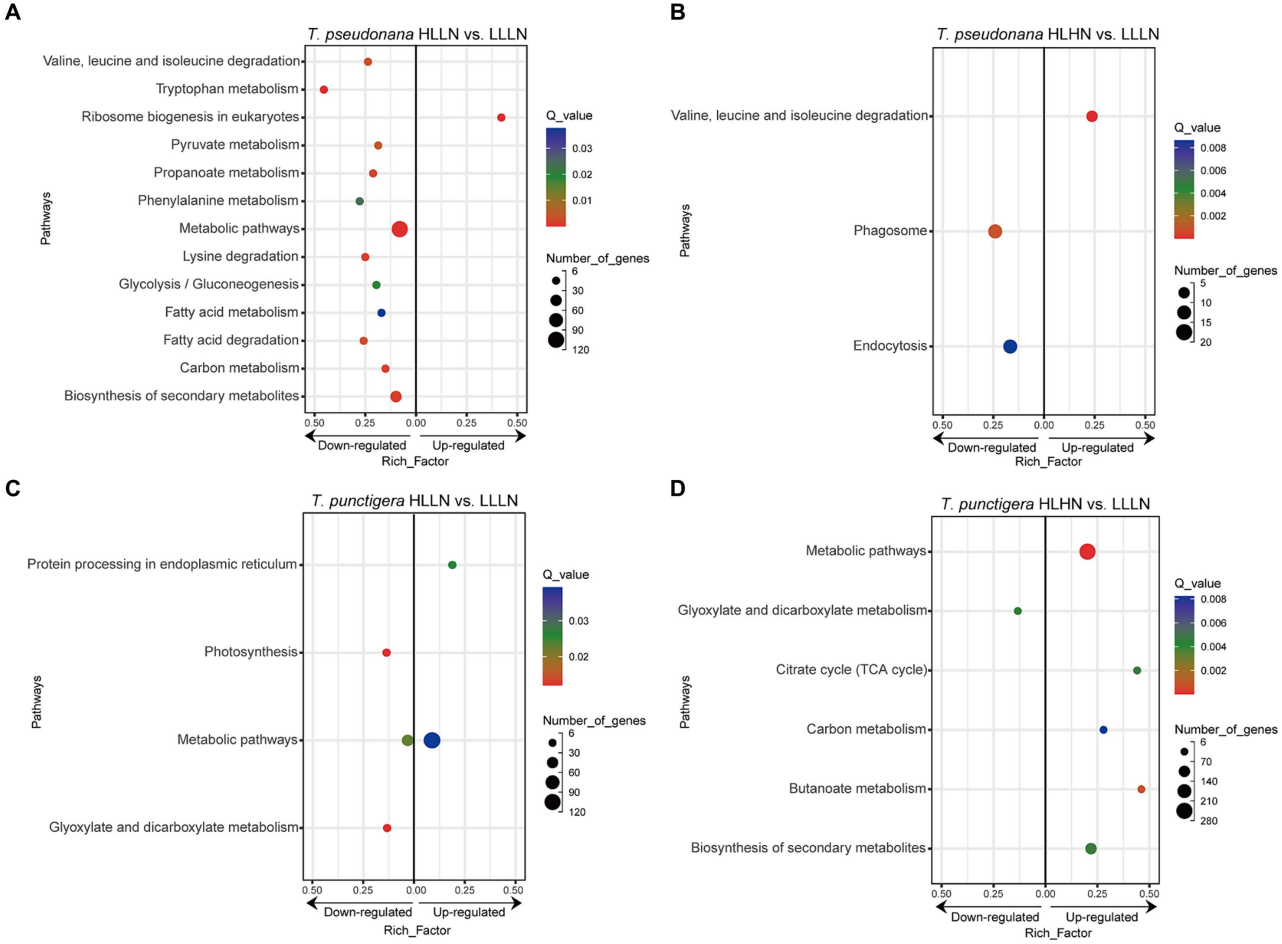
KEGG enrichment of differentially expressed genes (DEGs) under **(A)** high light low nitrogen (HLLN) vs. low light low nitrogen (LLLN) culture conditions for the small-celled *Thalassiosira pseudonana* and **(B)** high light high nitrogen (HLHN) vs. LLLN culture conditions for *T. pseudonana*, **(C)** HLLN vs. LLLN culture conditions for the lavrge-celled *T. punctigera* and **(D)** HLHN vs. LLLN culture conditions for *T. punctigera*. “a vs. b” means a compared to b. Functional categories of DEGs are grouped at KEGG level three. The rich factor for each pathway is plotted on the *x*-axes. In each graph, leftward and rightward dots indicated the down-regulated and up-regulated pathways, respectively. The DEGs were determined using DESeq2 with |log_2_(fold change)| >1 and *p* < 0.05. Only significantly enriched KEGG pathways (*Q*-value <0.05) were plotted.

**Figure 4:**
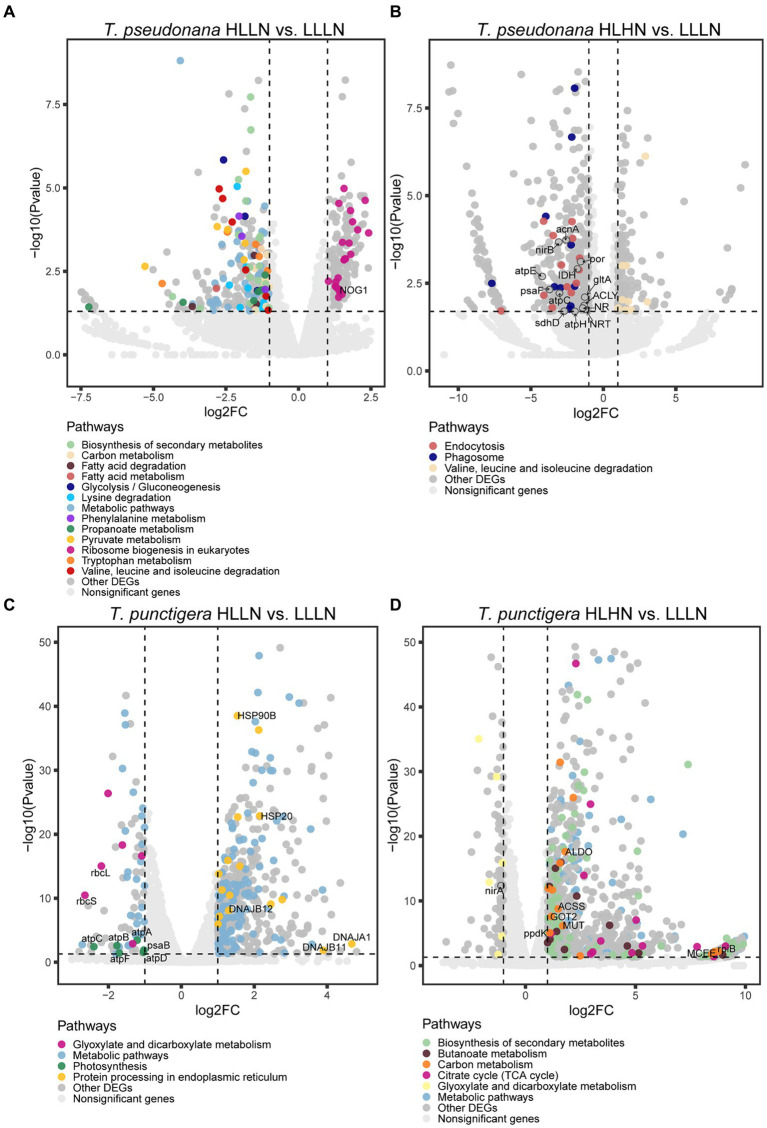
Volcano plots of genes in each pathway for the small-celled *Thalassiosira pseudonana* under **(A)** high light low nitrogen (HLLN) vs. low light low nitrogen (LLLN) culture conditions and **(B)** high light high nitrogen (HLHN) vs. low light low nitrogen (LLLN) culture conditions for *T. pseudonana*; and for the large-celled *T. punctigera* under **(C)** high light low nitrogen (HLLN) vs. low light low nitrogen (LLLN) culture conditions and **(D)** high light high nitrogen (HLHN) vs. low light low nitrogen (LLLN) culture conditions. “a vs. b” means a compared to b. Genes belonging to the significantly enriched KEGG pathways (level three, *Q*-value <0.05) were plotted in colors. The gene names in the enriched pathways are listed in Supplementary Tables S6–S9 for each algal species and treatment.

For *T. pseudonana*, 504 genes and 604 genes exhibited differential expressions in HLLN and HLHN relative to LLLN, respectively. Most genes were down-regulated under both comparisons (Figure 2A and Supplementary Tables S2, S5). KEGG pathway enrichment analyses showed that, when *T. pseudonana* culture conditions change from LLLN to HLLN, 12 pathways were significantly down-regulated and only ribosome biogenesis in the eukaryotes pathway was up-regulated (Figure 3A). *NOG1* gene in the ribosome biogenesis pathway is reported to be involved in the DNA mismatch repair system (Figure 4A and Supplementary Table S6). Four of the twelve depleted pathways are part of the amino acid metabolism: lysine degradation, phenylalanine metabolism, tryptophan metabolism, and valine, leucine and isoleucine degradation (Figure 3A). The remaining depleted pathways were related to carbohydrate metabolism (propanoate metabolism, pyruvate metabolism and glycolysis/gluconeogenesis), fatty acid metabolism/degradation, and other metabolism pathways (Figure 3A). While changing to HLHN conditions, the phagosome and endocytosis pathways were significantly down-regulated and only the valine, leucine and isoleucine degradation pathways were up-regulated for *T. pseudonana* when compared to LLLN conditions (Figure 3B). The PSI reaction center subunit III encoding gene (*psaF*) and adenosine triphosphate (ATP) synthase subunits encoding genes (*atpC*, *atpE*, *atpH*) were also significantly down regulated (Figure 4B). Tricarboxylic acid (TCA) cycle-related genes, including citrate synthase (*gltA*), ATP citrate lyase (*ACLY*), pyruvate:ferredoxin oxidoreductase (*por*), isocitrate dehydrogenase (*icd*), aconitase (*acnA*) and succinate dehydrogenase complex subunit D (*sdhD*) were significantly down-regulated (Figure 4B). Nitrogen metabolism-related genes, such as nitrate/nitrite transporter (*NRT*), nitrate reductase (*NR*) and nitrite reductase (*nirB*), were significantly down-regulated (Figure 4B).

Regarding the larger-celled *T. punctigera*, 555 genes and 966 genes exhibited differential expressions under HLLN and HLHN relative to LLLN conditions, and over 78 and 90% of the genes were up-regulated in HLLN and HLHN conditions, respectively (Figure 2B and Supplementary Tables S4, S7). The enrichment analyses showed that general metabolic pathways were enriched in both directions (Figure 3C), while the photosynthesis, glyoxylate and dicarboxylate metabolism pathways were down-regulated and only protein processing in the endoplasmic reticulum pathway was up-regulated under HLLN conditions (Figure 3C). In the photosynthesis pathway, one apoprotein encoding gene *psaB* and five chloroplast ATP synthase subunits encoding genes (*atpA*, *atpB*, *atpC*, *atpD*, *atpF*) were significantly down-regulated (Figure 4C and Supplementary Table S8). Also, the subunits of ribulose bisphosphate carboxylase encoding genes *rbcS* and *rbcL* that are involved in the glyoxylate and dicarboxylate metabolism pathways were also significantly down-regulated (Figure 4C and Supplementary Table S8). Regarding protein processing in the endoplasmic reticulum pathway, five heat shock proteins (HSPs) encoding genes (*DNAJB11*, *DNAJA1*, *DNAJB12*, *HSP20* and *HSP90B*) were significantly up-regulated (Figure 4C and Supplementary Table S8). When comparing HLHN to LLLN conditions, five pathways were significantly up-regulated, including butanoate, carbon, general (shown as metabolic pathways) and two carbohydrate metabolism pathways (i.e., the pathways for biosynthesis of secondary metabolites and the TCA cycle) (Figure 3D). In the carbon metabolism pathway, *MCEE*, *rpiB*, *ppdK*, *MUT*, *GOT2*, *ACSS*, *ALDO* and *maeB* were annotated as key genes in carbon fixation (Figure 4D and Supplementary Table S9). The glyoxylate and dicarboxylate metabolism pathways were significantly down-regulated under HLHN conditions (Figure 3D).

## Discussion

Light and nitrogen availability are recognized as prime drivers affecting diatom growth and stoichiometry (Cointet et al., 2019). In our study, *T. pseudonana* and *T. punctigera* employed contrasting strategies to cope with light and nitrogen stresses, even though the two species are classified in the same genus. When changing from LLLN to HLLN, diatoms were switched to high light maintaining low nitrogen stress. The growth rate of the small-celled *T. pseudonana* remained unchanged with the switch, while that of the large-celled *T. punctigera* decreased significantly (Figure 1). Studies have demonstrated that high light exposure can cause photodamage, limiting diatom growth (Wu et al., 2011; Dong et al., 2016), and this appears to have been the case for *T. punctigera*. The transcriptome profile reveals that photosynthesis pathways were significantly down-regulated for *T. punctigera* (Figure 3C). To be specific, the expression of the *psaB* gene and the chloroplast ATP synthase subunits encoding genes (*atpA*, *atpB*, *atpC*, *atpD*, *atpF*) decreased in expression (Figure 4C). The *psaB* gene encoding apoprotein is one of the reaction center subunits of photosystem I (PS I), which binds the primary electron donor of PSI (P700), as well as the chlorophyll (A0), phylloquinone (A1) and iron–sulphur (FX) electron acceptors (Golbeck, 1987). The genes encoding the ATP synthase proteins use the electrochemical proton gradient generated by photosynthesis for their ATP production (Hahn et al., 2018). Consequently, the electron transfer and ATP generation efficiency were suppressed in the photosystem. Corresponding to down-regulation of the photosynthesis pathway, *rbcS* and *rbcL* genes, involved in glyoxylate and dicarboxylate metabolism, were also down-regulated (Figure 4C). These two genes encode the subunits of ribulose bisphosphate carboxylase, which are closely related to carbon fixation in the photosystem (Sen et al., 2011; Yamada et al., 2019). Thus, the down regulation of the *psaB*, *atpA*, *atpB*, *atpC*, *atpD*, *atpF*, *rbcS* and *rbcL* genes collectively decreases electron transfer efficiency, ATP generation, carbon fixation rates, and, ultimately, the growth of *T. punctigera*. Up-regulation was observed in the Hsp40/DnaJ protein encoding genes (*DNAJB11*, *DNAJA1*, *DNAJB12*) and the *HSP20* and *HSP90B* genes (Figure 4C). HSPs are widely distributed in cells and take part in a variety of processes to keep cell integrity, maintain protein homeostasis, and respond to stresses (Hu et al., 2022). Studies have shown that Hsp40/DnaJ proteins are involved in the optimization of photosynthetic reactions, stabilization of the photosystem II (PSII) complex under high light stress, and reduction of reactive oxygen species (ROS) accumulation in plants (Kong et al., 2014; Liu et al., 2023). Therefore, up-regulation of Hsp40/DnaJ proteins would suggest that *T. punctigera* was attempting to protect itself from high light exposure. We conclude that *T. punctigera* was photodamaged in this treatment, evidenced by the decreased growth rate and modified transcriptomic pathways.

Small *T. pseudonana*, in contrast, remained at the same growth rate when changing from LLLN to HLLN (Figure 1). The transcriptome profile reveals that 12 pathways were significantly down-regulated (Figure 3A), while only the ribosome biogenesis-in-eukaryotes pathway was significantly up-regulated (Figure 3B). Ribosome biogenesis is fundamental to most cellular processes, mainly involving the synthesis of rRNAs and ribosomal proteins, which are required for proliferation and cell division (Kumar, 2021). However, the significant up-regulation of ribosome biogenesis is not reflected in an unchanging growth rate. We speculate that the up-regulation of ribosome biogenesis is a response to photodamage under HLLN conditions. With a high surface area-to-volume ratio, small-celled *T. pseudonana* have a higher light absorption rate per unit of chlorophyll, rendering cells more vulnerable to high light exposure (Finkel et al., 2010). Studies have shown that ribosome biogenesis and DNA repair processes are tightly connected (Ogawa and Baserga, 2017). For example, the *NOG1* gene was significantly up-regulated in small-celled *T. pseudonana* under HLLN conditions (Figure 4A). It encodes a multi-functional protein and was reported to be involved in the DNA mismatch repair system (Xue et al., 2023). Moreover, a recent study revealed that the assembly of extra ribosomes will increase the translation capacity and improve turnover of plastid-encoded photosystems subunits, which is critical for algal acclimation to high light conditions (Djouani-Tahri et al., 2022). Thus, up-regulation of ribosome biogenesis could help small-celled *T. pseudonana* cope with high light stress. Ribosome biogenesis is an energy-consuming biosynthetic process, and about 60% of a cell’s energy is spent on ribosome production and maintenance (Kumar, 2021). It is reasonable that other pathways are down-regulated to conserve energy and matter, especially in small diatom cells with low nutrient storage capacity (Finkel et al., 2010). Notably, four of the down-regulated pathways were for amino acid production (Figure 3A). It is possible that small-celled *T. pseudonana* were experiencing nitrogen deficiency stress. Amino acids are a fundamental necessity for all life. For example, the aromatic amino acids, phenylalanine and tryptophan, serve as building blocks for many compounds essential to plant structure, reproduction, defense and communication (Tzin and Galili, 2010). Lysine not only functions as a building block of proteins, but is also an important signal, interacting with other metabolic fluxes (Galili, 2002). When nitrogen availability is low, photosynthetic organisms experience a shortage of the ammonium used for amino acid biosynthesis (Lea and Miflin, 2018). Thus, slowing down the degradation or transformation of amino acids should protect diatoms from photosynthesis dysfunction (Chen et al., 2022). We conclude that *T. pseudonana* sacrificed cellular processes, especially amino acid metabolism, to reallocate resources to combat photodamage under HLLN conditions.

Changing from LLLN to HLHN conditions, diatoms experienced increased light and nitrogen resources. This mimics conditions following the passage of a tropical cyclone, where the water column is well-mixed and diatoms are brought to the lit surface layer. Physiological experiments confirm that the large-celled *T. punctigera* prefer these high light and high nitrogen conditions (Figure 1). Corresponding to the increased growth rate under HLHN conditions, the TCA cycle of the large-celled *T. punctigera* was significantly up-regulated (Figure 3D). The TCA cycle is the fundamental pathway for the oxidation of carbohydrates, proteins and fatty acids to generate ATP, which provides energy for cellular development, growth and reproduction (Zhang and Fernie, 2018). In photosynthetic eukaryotes, the TCA cycle is also the major player in carbon fixation (Bar-Even et al., 2010). Indeed, the up-regulated genes *MCEE*, *rpiB*, *ppdK*, *MUT*, *GOT2*, *ACSS*, *ALDO*, and *maeB* involved in carbon metabolism were annotated as key genes in carbon fixation (Figure 4D and Supplementary Table S3), which provides the carbon resources required by the anabolic metabolism (Bar-Even et al., 2010). In addition, the TCA cycle is deeply nested within many other essential cellular processes and has numerous auxiliary functions including photosynthesis optimization, carbohydrate metabolism, carbon–nitrogen interactions, signaling, and others (Zhang and Fernie, 2023). Carbon metabolism, butanoate metabolism and general metabolism pathways were significantly up-regulated in *T. punctigera* (Figure 3D). Therefore, the transcriptome profile revealed that adding nitrate enabled *T. punctigera* to compensate for damage from high light exposure. The importance of nitrogen for microalgal photosynthetic production is well-established in the general sense (Turpin et al., 1988; Evans and Clarke, 2019). In our results, we found that the ferredoxin-nitrite reductase encoding gene (*nirA*) was down-regulated under HLHN relative to LLLN conditions, which suggests that *T. punctigera* was finding ample nitrogen, causing nitrate assimilation to slow down (Burger et al., 1991). Results indicate that adding nitrate under high light conditions promotes growth and potential algal bloom formation in *T. punctigera*.

Contrary to the fast-growing *T. punctigera*, the growth rate of the small-celled *T. pseudonana* slightly increased under HLHN conditions but was not significantly different from the LLLN treatment (Figure 1). KEGG pathway enrichment analysis showed that both the phagosome and endocytosis pathways were significantly down-regulated (Figure 3B). The phagosome and endocytosis pathways are cellular processes in which substances are directly brought into the cell (Miaczynska and Stenmark, 2008). Although, the two pathways have multiple cellular functions including cellular growth, development, signaling, and nutrient delivery, both the phagosome and endocytosis mechanisms are reported as central to the establishment and maintenance of cell homeostasis in plants and algae (Craddock and Yang, 2012; Levin et al., 2016). Significant down-regulation of the phagosome and endocytosis pathways may indicate that *T. pseudonana* was not heavily stressed in the HLHN conditions. While, the constant growth rate in HLHN relative to LLLN conditions could be explained by the great number of down-regulated genes. In the HLHN treatment, 875 of 966 DEGs were down-regulated relative to LLLN conditions, indicating that a lot of cellular processes are slowing down. Even though most down-regulated genes were not significantly enriched on specific pathways, genes that participate in photosynthesis and the TCA cycle were significantly down-regulated (Figure 4B). For example, the PSI reaction center subunit III encoding gene (*psaF*) and the ATP synthase subunits encoding genes (*atpC*, *atpE*, *atpH*) were significantly down-regulated (Figure 4B), suggesting that electron transfer and ATP synthase efficiency in the photosynthetic system have diminished (Wöstemeyer and Oelmüller, 2003; Hahn et al., 2018). As mentioned before, small cells are more vulnerable to high light exposure (Finkel et al., 2010), so that *T. pseudonana* was under high light stress in HLHN treatment. The genes encoding citrate synthase (*gltA*), ATP citrate lyase (*ACLY*), pyruvate:ferredoxin oxidoreductase (*por*), isocitrate dehydrogenase (*icd*), aconitase (*acnA*) and succinate dehydrogenase complex subunit D (*sdhD*) were all significantly down-regulated, and these genes are essential participants in the TCA cycle (Park et al., 1994; Baysal et al., 2000; Hartong et al., 2008; Ikeda et al., 2010; Chypre et al., 2012; Michta et al., 2014). The depressed expression of the genes, essential to the photosynthetic system and the TCA cycle, would suppress diatom growth and reproduction. Also, the genes encoding nitrate/nitrite transporter (*NRT*), nitrate reductase (*NR*) and nitrite reductase (*nirB*) were significantly down-regulated in the HLHN treatment (Figure 4B), indicating that *T. pseudonana* had sufficient nitrogen, resulting in a slowdown in nitrate/nitrite import and assimilation (Wang and Gunsalus, 2000; Wang et al., 2009; Coyne, 2010). In contrast to *T. punctigera*, high light exposure was the sole critical factor for *T. pseudonana* and the supplement of nitrate did not compensate for the light stress damage. We conclude that the small-celled *T. pseudonana* is vulnerable to light stress and less likely to form an algal bloom under HLHN conditions.

## Conclusion

**Figure 5:**
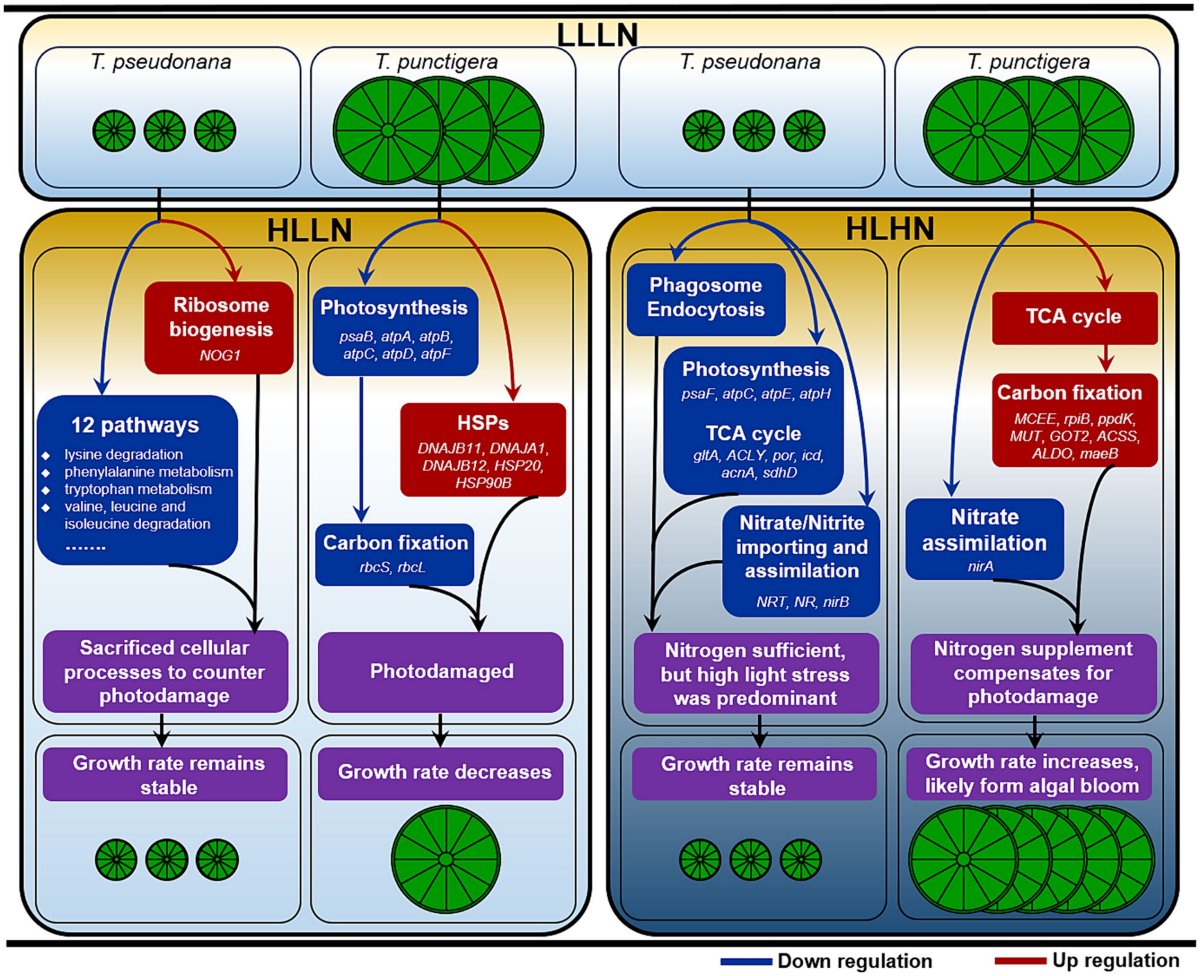
Schematic illustration of the key cellular processes of the two diatom species under high light low nitrogen (HLLN) and high light high nitrogen (HLHN) compared to low light low nitrogen (LLLN) conditions. Genes displayed in each box are DEGs of the corresponding pathways in that box. Red and blue rectangular boxes indicate up- and down-regulation, respectively.

The two diatom species displayed quite different cellular responses in coping with changes to their light and nitrogen levels (Figure 5). When the diatoms were transferred to HLLN conditions, the large-celled *T. punctigera* was photodamaged, indicated by down-regulation of photosynthesis pathway and carbon fixation-related genes. The small-celled *T. pseudonana*, on the other hand, downregulated many cellular processes to conquer photodamage and keep growth rates similar when switched from LLLN to HLLN conditions. When changing to HLHN conditions, the supplement of nitrogen allowed the large-celled *T. punctigera* to compensate for the photodamage inflicted by more light by boosting the TCA cycle and carbon fixation. Consequently, the growth rate of the large-celled *T. punctigera* increased under HLHN conditions, which indicated that they could form blooms after tropical storm. However, the impact of high light stress on the small-celled *T. pseudonana* was too great for nitrate supplementation to compensate for the photodamage. We conclude that the small-celled *T. pseudonana* is more vulnerable to high light stress, while the large-celled *T. punctigera* is more likely to form algal blooms after storms.

## Data Availability

The datasets presented in this study can be found in online repositories. The names of the repository/repositories and accession number(s) can be found in the article/Supplementary material.

## References

[ref1] Bar-Even A. Noor E. Lewis N. E. Milo R. (2010). Design and analysis of synthetic carbon fixation pathways. Proc. Natl. Acad. Sci. U. S. A. 107, 8889–8894. doi: 10.1073/pnas.0907176107, PMID: 20410460 PMC2889323

[ref2] Baysal B. E. Ferrell R. E. Willett-Brozick J. E. Lawrence E. C. Myssiorek D. Bosch A.. (2000). Mutations in *SDHD*, a mitochondrial complex II gene, in hereditary paraganglioma. Science 287, 848–851. doi: 10.1126/science.287.5454.848 10657297

[ref3] Berges J. A. Falkowski P. G. (1998). Physiological stress and cell death in marine phytoplankton: induction of proteases in response to nitrogen or light limitation. Limnol. Oceanogr. 43, 129–135. doi: 10.4319/lo.1998.43.1.0129

[ref4] Brodie J. Schroeder T. Rohde K. Faithful J. Masters B. Dekker A.. (2010). Dispersal of suspended sediments and nutrients in the great barrier reef lagoon during river-discharge events: conclusions from satellite remote sensing and concurrent flood-plume sampling. Mar. Freshw. Res. 61:651. doi: 10.1071/MF08030

[ref5] Burger G. Tilburn J. Scazzocchio C. (1991). Molecular cloning and functional characterization of the pathway-specific regulatory gene nirA, which controls nitrate assimilation in aspergillus nidulans. Mol. Cell. Biol. 11, 795–802. doi: 10.1128/MCB.11.2.795, PMID: 1990284 PMC359731

[ref6] Chen L.-H. Cheng Z.-X. Xu M. Yang Z.-J. Yang L.-T. (2022). Effects of nitrogen deficiency on the metabolism of organic acids and amino acids in *Oryza sativa*. Plan. Theory 11:2576. doi: 10.3390/plants11192576, PMID: 36235442 PMC9572205

[ref7] Chypre M. Zaidi N. Smans K. (2012). ATP-citrate lyase: a mini-review. Biochem. Biophys. Res. Commun. 422, 1–4. doi: 10.1016/j.bbrc.2012.04.144, PMID: 22575446

[ref8] Cointet E. Wielgosz-Collin G. Bougaran G. Rabesaotra V. Gonçalves O. Méléder V. (2019). Effects of light and nitrogen availability on photosynthetic efficiency and fatty acid content of three original benthic diatom strains. PLoS One 14:e0224701. doi: 10.1371/journal.pone.0224701, PMID: 31694047 PMC6834396

[ref9] Coyne K. J. (2010). Nitrate reductase (NR1) sequence and expression in the harmful alga heterosigma akashiwo (raphidophyceae)1: heterosigma nitrate reductase. J. Phycol. 46, 135–142. doi: 10.1111/j.1529-8817.2009.00781.x

[ref10] Craddock C. Yang Z. (2012). Endocytic signaling pathways in leaves and roots; same players different rules. Front. Plant Sci. 3:219. doi: 10.3389/fpls.2012.00219, PMID: 23060890 PMC3462323

[ref11] Djouani-Tahri E. B. Nellaepalli S. Auroy P. Billon E. Burlacot A. Chaux-Jukic F.. (2022). Boosting chloroplast ribosome biogenesis by a plastidial DEAD-box RNA helicase is critical for high light acclimation. Plant Biol. doi: 10.1101/2022.05.16.492170

[ref12] Dong H.-P. Dong Y.-L. Cui L. Balamurugan S. Gao J. Lu S.-H.. (2016). High light stress triggers distinct proteomic responses in the marine diatom *Thalassiosira pseudonana*. BMC Genomics 17:994. doi: 10.1186/s12864-016-3335-5, PMID: 27919227 PMC5139114

[ref13] Evans J. R. Clarke V. C. (2019). The nitrogen cost of photosynthesis. J. Exp. Bot. 70, 7–15. doi: 10.1093/jxb/ery366 30357381

[ref14] Finkel Z. V. Beardall J. Flynn K. J. Quigg A. Rees T. A. V. Raven J. A. (2010). Phytoplankton in a changing world: cell size and elemental stoichiometry. J. Plankton Res. 32, 119–137. doi: 10.1093/plankt/fbp098

[ref15] Galili G. (2002). N EW I NSIGHTS INTO THE r EGULATION AND f UNCTIONAL s IGNIFICANCE OF l YSINE m ETABOLISM IN p LANTS. Annu. Rev. Plant Biol. 53, 27–43. doi: 10.1146/annurev.arplant.53.091401.110929 12221976

[ref16] Golbeck J. H. (1987). Structure, function and organization of the photosystem I reaction center complex. Biochim. Biophys. Acta 895, 167–204. doi: 10.1016/S0304-4173(87)80002-2, PMID: 3333014

[ref17] Hahn A. Vonck J. Mills D. J. Meier T. Kühlbrandt W. (2018). Structure, mechanism, and regulation of the chloroplast ATP synthase. Science 360:eaat4318. doi: 10.1126/science.aat4318, PMID: 29748256 PMC7116070

[ref18] Hartong D. T. Dange M. McGee T. L. Berson E. L. Dryja T. P. Colman R. F. (2008). Insights from retinitis pigmentosa into the roles of isocitrate dehydrogenases in the Krebs cycle. Nat. Genet. 40, 1230–1234. doi: 10.1038/ng.223, PMID: 18806796 PMC2596605

[ref19] Hong G. Zhang W. Li H. Shen X. Guo Z. (2014). Separate enrichment analysis of pathways for up- and downregulated genes. J. R. Soc. Interface 11:20130950. doi: 10.1098/rsif.2013.0950, PMID: 24352673 PMC3899863

[ref20] Hu C. Yang J. Qi Z. Wu H. Wang B. Zou F.. (2022). Heat shock proteins: biological functions, pathological roles, and therapeutic opportunities. MedComm 3:e161. doi: 10.1002/mco2.161, PMID: 35928554 PMC9345296

[ref21] Ikeda T. Yamamoto M. Arai H. Ohmori D. Ishii M. Igarashi Y. (2010). Enzymatic and electron paramagnetic resonance studies of anabolic pyruvate synthesis by pyruvate: ferredoxin oxidoreductase from *Hydrogenobacter thermophilus*. FEBS J. 277, 501–510. doi: 10.1111/j.1742-4658.2009.07506.x, PMID: 20015072

[ref22] Jiang T. Wu G. Niu P. Cui Z. Bian X. Xie Y.. (2022). Short-term changes in algal blooms and phytoplankton community after the passage of super typhoon Lekima in a temperate and inner sea (Bohai Sea) in China. Ecotoxicol. Environ. Saf. 232:113223. doi: 10.1016/j.ecoenv.2022.113223, PMID: 35091297

[ref23] Kang J. Xie Y. Lin Y. Wang Y. (2022). Algal bloom, succession, and drawdown of silicate in the Chukchi Sea in summer 2010. Ecosystems 25, 320–336. doi: 10.1007/s10021-021-00657-1

[ref24] Kong F. Deng Y. Zhou B. Wang G. Wang Y. Meng Q. (2014). A chloroplast-targeted DnaJ protein contributes to maintenance of photosystem II under chilling stress. J. Exp. Bot. 65, 143–158. doi: 10.1093/jxb/ert357, PMID: 24227338 PMC3883286

[ref25] Kumar V. (2021). “Ribosomal biogenesis in eukaryotes” in Emerging concepts in ribosome structure, biogenesis, and function (Amsterdam. Elsevier), 129–150.

[ref26] Lea P. J. Miflin B. J. (2018). “Nitrogen assimilation and its relevance to crop improvement” in Annual plant reviews online. ed. Roberts J. A. (Hoboken: Wiley), 1–40.

[ref27] Levin R. Grinstein S. Canton J. (2016). The life cycle of phagosomes: formation, maturation, and resolution. Immunol. Rev. 273, 156–179. doi: 10.1111/imr.12439, PMID: 27558334

[ref28] Li G. Campbell D. A. (2017). Interactive effects of nitrogen and light on growth rates and RUBISCO content of small and large centric diatoms. Photosynth. Res. 131, 93–103. doi: 10.1007/s11120-016-0301-7, PMID: 27566625 PMC5167766

[ref29] Litchman E. Klausmeier C. A. Yoshiyama K. (2009). Contrasting size evolution in marine and freshwater diatoms. Proc. Natl. Acad. Sci. U. S. A. 106, 2665–2670. doi: 10.1073/pnas.0810891106, PMID: 19202058 PMC2650323

[ref30] Liu S. Mo X. Sun L. Gao L. Su L. An Y.. (2023). MsDjB4, a HSP40 chaperone in alfalfa (*Medicago sativa* L.), improves alfalfa hairy root tolerance to aluminum stress. Plan. Theory 12:2808. doi: 10.3390/plants12152808, PMID: 37570962 PMC10421020

[ref31] Ma X. Jacoby C. A. Johnson K. B. (2021). Grazing by the copepod *Parvocalanus crassirostris* on Picochlorum sp. at Harmful bloom densities and the role of particle size. Front. Mar. Sci. 8:664154. doi: 10.3389/fmars.2021.664154

[ref32] Ma X. Johnson K. B. Gu B. Zhang H. Li G. Huang X.. (2022). The in-situ release of algal bloom populations and the role of prokaryotic communities in their establishment and growth. Water Res. 219:118565. doi: 10.1016/j.watres.2022.118565, PMID: 35597219

[ref33] Malviya S. Scalco E. Audic S. Vincent F. Veluchamy A. Poulain J.. (2016). Insights into global diatom distribution and diversity in the world’s ocean. Proc. Natl. Acad. Sci. U. S. A. 113, E1516–E1525. doi: 10.1073/pnas.1509523113, PMID: 26929361 PMC4801293

[ref34] Maslać N. Sidhu C. Teeling H. Wagner T. (2022). Comparative transcriptomics sheds light on remodeling of gene expression during Diazotrophy in the thermophilic methanogen *Methanothermococcus thermolithotrophicus*. MBio 13:e0244322. doi: 10.1128/mbio.02443-22, PMID: 36409126 PMC9765008

[ref35] Miaczynska M. Stenmark H. (2008). Mechanisms and functions of endocytosis. J. Cell Biol. 180, 7–11. doi: 10.1083/jcb.200711073, PMID: 18195098 PMC2213624

[ref36] Michta E. Ding W. Zhu S. Blin K. Ruan H. Wang R.. (2014). Proteomic approach to reveal the regulatory function of Aconitase AcnA in oxidative stress response in the antibiotic producer *Streptomyces viridochromogenes* Tü494. PLoS One 9:e87905. doi: 10.1371/journal.pone.0087905, PMID: 24498397 PMC3912134

[ref37] Ogawa L. M. Baserga S. J. (2017). Crosstalk between the nucleolus and the DNA damage response. Mol. BioSyst. 13, 443–455. doi: 10.1039/C6MB00740F, PMID: 28112326 PMC5340083

[ref38] Okhapkin A. G. Genkal S. I. Vodeneeva E. L. Sharagina E. M. Bondarev O. O. (2016). To ecology and morphology of *Thalassiosira incerta* Makarova (Bacillariophyta). Inland Water Biol. 9, 126–134. doi: 10.1134/S1995082916020139

[ref39] Park J. S. Jung S. W. Lee S. D. Yun S. M. Lee J. H. (2016). Species diversity of the genus *Thalassiosira* (Thalassiosirales, Bacillariophyta) in South Korea and its biogeographical distribution in the world. Phycologia 55, 403–423. doi: 10.2216/15-66.1

[ref40] Park S. J. McCabe J. Turna J. Gunsalus R. P. (1994). Regulation of the citrate synthase (gltA) gene of *Escherichia coli* in response to anaerobiosis and carbon supply: role of the arcA gene product. J. Bacteriol. 176, 5086–5092. doi: 10.1128/jb.176.16.5086-5092.1994, PMID: 8051021 PMC196348

[ref41] Qin Z. Xia X. Mai G. Tan Y. Li G. (2021). Differential physiological responses of small Thalassiosira pseudonana and large *Thalassiosira punctigera* to the shifted-high light and nitrogen. JMSE 9:450. doi: 10.3390/jmse9050450

[ref42] Sen L. Fares M. A. Liang B. Gao L. Wang B. Wang T.. (2011). Molecular evolution of rbcL in three gymnosperm families: identifying adaptive and coevolutionary patterns. Biol. Direct 6:29. doi: 10.1186/1745-6150-6-29, PMID: 21639885 PMC3129321

[ref43] Sidabutar T. Srimariana E. S. Wouthuyzen S. (2021). Phytoplankton species potentially Harmful algal blooms (HABs) in Jakarta Bay. IOP Conf. Ser. Earth Environ. Sci. 744:012077. doi: 10.1088/1755-1315/744/1/012077

[ref44] Smith D. L. Johnson K. B. (1996). A guide to marine coastal plankton and marine invertebrate larvae. 2nd Edn. Dubuque, Iowa: Kendall/Hunt Pub. Co.

[ref45] Solovchenko A. E. Khozin-Goldberg I. Didi-Cohen S. Cohen Z. Merzlyak M. N. (2008). Effects of light intensity and nitrogen starvation on growth, total fatty acids and arachidonic acid in the green microalga Parietochloris incisa. J. Appl. Phycol. 20, 245–251. doi: 10.1007/s10811-007-9233-0

[ref46] Tamames J. Puente-Sánchez F. (2019). SqueezeMeta, a highly portable, fully automatic metagenomic analysis pipeline. Front. Microbiol. 9:3349. doi: 10.3389/fmicb.2018.03349, PMID: 30733714 PMC6353838

[ref47] Turpin D. H. Elrifi I. R. Birch D. G. Weger H. G. Holmes J. J. (1988). Interactions between photosynthesis, respiration, and nitrogen assimilation in microalgae. Can. J. Bot. 66, 2083–2097. doi: 10.1139/b88-286

[ref48] Tzin V. Galili G. (2010). New insights into the shikimate and aromatic amino acids biosynthesis pathways in plants. Mol. Plant 3, 956–972. doi: 10.1093/mp/ssq048, PMID: 20817774

[ref49] Ulgen E. Ozisik O. Sezerman O. U. (2019). pathfindR: An R package for comprehensive identification of enriched pathways in omics data through active subnetworks. Front. Genet. 10:858. doi: 10.3389/fgene.2019.00858, PMID: 31608109 PMC6773876

[ref50] von Dassow P. Petersen T. W. Chepurnov V. A. Virginia Armbrust E. (2008). Inter- and intraspecific relationships between nuclear DNA content and cell size in selected members of the centric diatom genus *Thalassiosira* (Bacillariophyceae). J. Phycol. 44, 335–349. doi: 10.1111/j.1529-8817.2008.00476.x, PMID: 27041190

[ref51] Wang H. Gunsalus R. P. (2000). The *nrfA* and *nirB* nitrite reductase operons in *Escherichia coli* are expressed differently in response to nitrate than to nitrite. J. Bacteriol. 182, 5813–5822. doi: 10.1128/JB.182.20.5813-5822.2000, PMID: 11004182 PMC94705

[ref52] Wang R. Xing X. Wang Y. Tran A. Crawford N. M. (2009). A genetic screen for nitrate regulatory mutants captures the nitrate transporter gene *NRT1.1*. Plant Physiol. 151, 472–478. doi: 10.1104/pp.109.140434, PMID: 19633234 PMC2735993

[ref53] Wöstemeyer A. Oelmüller R. (2003). The promoter of the spinachPsaF gene for the subunit III of the photosystem I reaction center directs β-glucuronidase gene expression in transgenic tobacco roots. J. Plant Physiol. 160, 503–508. doi: 10.1078/0176-1617-00912 12806778

[ref54] Wu H. Cockshutt A. M. McCarthy A. Campbell D. A. (2011). Distinctive photosystem II Photoinactivation and protein dynamics in marine diatoms. Plant Physiol. 156, 2184–2195. doi: 10.1104/pp.111.178772, PMID: 21617029 PMC3149953

[ref55] Xia X. Lee P. Cheung S. Lu Y. Liu H. (2020). Discovery of Euryhaline Phycoerythrobilin-containing *Synechococcus* and its mechanisms for adaptation to estuarine. Environments 5:e00842-20. doi: 10.1128/mSystems.00842-20, PMID: 33323414 PMC7771541

[ref56] Xue Q. Zhu Z. Xue Z. Yang F. Cao W. Liu X.. (2023). NOG1 downregulates type I interferon production by targeting phosphorylated interferon regulatory factor three. PLoS Pathog. 19:e1011511. doi: 10.1371/journal.ppat.1011511, PMID: 37410776 PMC10353805

[ref57] Yamada K. Davydov I. I. Besnard G. Salamin N. (2019). Duplication history and molecular evolution of the rbcS multigene family in angiosperms. J. Exp. Bot. 70, 6127–6139. doi: 10.1093/jxb/erz363, PMID: 31498865 PMC6859733

[ref58] Zhang Y. Fernie A. R. (2018). On the role of the tricarboxylic acid cycle in plant productivity. JIPB 60, 1199–1216. doi: 10.1111/jipb.12690 29917310

[ref59] Zhang Y. Fernie A. R. (2023). The role of TCA cycle enzymes in plants. Adv. Biol. 7:2200238. doi: 10.1002/adbi.202200238 37341441

